# Quality of care and patient safety at healthcare institutions in Oman: quantitative study of the perspectives of patients and healthcare professionals

**DOI:** 10.1186/s12913-021-07152-2

**Published:** 2021-10-16

**Authors:** Fatma Al-Jabri, Tarja Kvist, Reijo Sund, Hannele Turunen

**Affiliations:** 1 Department of Nursing Science, University of Eastern Finland, P.O. Box 1627, 70211 Kuopio, Finland; 2 Institute of Clinical Medicine, School of Medicine, Faculty of Health Sciences, University of Eastern Finland, P.O. Box 1627, 70211 Kuopio, Finland

**Keywords:** Patients’ perspectives, Healthcare professionals’ perspectives, Quality of care, Patient safety, Quantitative study, Oman

## Abstract

**Background:**

Oman’s healthcare system has rapidly transformed in recent years. A recent Report of Quality and Patient Safety has nevertheless highlighted decreasing levels of patient safety and quality culture among healthcare professionals. This indicates the need to assess the quality of care and patient safety from the perspectives of both patients and healthcare professionals.

**Objectives:**

This study aimed to examine (1) patients’ and healthcare professionals’ perspectives on overall quality of care and patient safety standards at two tertiary hospitals in Oman and (2) which demographic characteristics are related to the overall quality of care and patient safety.

**Methods:**

A cross-sectional study design was employed. Data were collected by two items: overall quality of care and patient safety, incorporated in the Revised Humane Caring Scale, and Healthcare Professional Core Competency Instrument. Questionnaires were distributed to (1) patients (*n* = 600) and (2) healthcare professionals (nurses and physicians) (*n* = 246) in three departments (medical, surgical and obstetrics and gynaecology) at two tertiary hospitals in Oman towards the end of 2018 and the beginning of 2019. Descriptive statistics and binary logistic regression were used for data analysis.

**Results:**

A total of 367 patients and 140 healthcare professionals completed the questionnaires, representing response rates of 61.2% and 56.9%, respectively. Overall, quality of care and patient safety were perceived as high, with the healthcare professionals rating quality of care (M = 4.36; SD = 0.720) and patient safety (M = 4.39; SD = 0.675) slightly higher than the patients did (M = 4.23; SD = 0.706), (M = 4.22; SD = 0.709). The findings indicated an association between hospital variables and overall quality of care (OR = 0.095; 95% CI = 0.016–0.551; *p* = 0.009) and patient safety (OR = 0.153; 95% CI = 0.027–0.854; *p* = 0.032) among healthcare professionals. Additionally, an association between the admission/work area and participants’ perspectives on the quality of care (patients, OR = 0.257; 95% CI = 0.072–0.916; *p* = 0.036; professionals, OR = 0.093; 95% CI = 0.009–0.959; *p* = 0.046) was found.

**Conclusions:**

The perspectives of both patients and healthcare professionals showed that they viewed both quality of care and patient safety as excellent, with slight differences, indicating a high level of patient satisfaction and competent healthcare delivery professionals. Such perspectives can provide meaningful and complementary insights on improving the overall standards of healthcare delivery systems.

**Supplementary Information:**

The online version contains supplementary material available at 10.1186/s12913-021-07152-2.

## Background

Quality of care and patient safety are undoubtedly two distinctive targets for leading healthcare systems around the world [1–3]. These targets continue to be at the top of the agenda for healthcare regulators and policy markers in the Sultanate of Oman [4]. Oman’s Ministry of Health (MOH) established the Department of Quality and Patient Safety in regional hospitals in 2007 to implement a quality assurance strategy [5]. It also adopted the Patient Safety Friendly Hospital Initiative (PSFHI) in 2015 to promote an inclusive and integrative healthcare system [6]. Such efforts have considerably improved the outcomes of the healthcare system, for instance, by drastically reducing mortality rates of children under five by 72% from 1990 to 2013 and maternal mortality rates by 55% from 1990 to 2013 [5, 7].

Although Oman’s healthcare system was ranked by the World Health Organization (WHO) as one of the10 best healthcare systems in the world in 2012 [8], a recent Report of Quality and Patient Safety (RQPS) highlighted a decreased level of patient safety and quality of care culture among healthcare professionals (HCPs) [9]. The report called for a comprehensive assessment of quality of care and patient safety to include the perspectives of both HCPs (as service providers) and patients (as service users). The report recognizes that HCPs typically focus on long-term and sustainable solutions while managing service and delivery costs [10]. Their core competencies and wider technical excellence - often play a pivotal role in the overall classification of quality of care and patient safety from the perspective of healthcare providers [3, 11–13].

On the other hand, patients tend to value short-term comforts [14]. Their perspectives are usually based on the overall healthcare system, practice type, and care providers’ personal and clinical skills [13, 15, 16]. This explains why world organizations such as the Council of Europe (CoE) [17], the WHO [3], and the United States (US) Institute of Medicine (IOM) [18] all enhance that patients’ views of quality care are important in addition to providers’ views to find the right balance between two perspectives and provide additional insight into areas where change is needed. Therefore, this study is part of a larger study that aims to (1) consolidate patients’ and HCPs’ (nurses and physicians) perspectives on quality of care and patient safety at two tertiary hospitals in Oman [19] and (2) identify the participant characteristics most related to quality of care and patient safety. The outcomes of this study will provide meaningful and complementary insights for improving the overall standards of healthcare delivery systems.

## Methods

### Study context

This study was conducted in Oman, a high-income Arab country of 4.6 million people; one- third of its population lives in the capital city of Oman [20]. It has experienced rapid economic and social transformation since 1970, which has resulted in better quality living standards. By 2019, Oman’s MOH had a total of 50 hospitals; 5049 beds; 269 health centres, clinics, and dispensaries (governmental); and 1254 private clinics. The total numbers of doctors and nurses were 6419 and 14,491, respectively. In 2019, for every 10,000 people, there were 21 doctors and 44 nurses in the country, and the nurse–doctor ratio was 2:1. The healthcare system in Oman is characterised by its universal coverage for both citizens and expatriates and its health system comprises both the government and private sectors. Healthcare is provided in facilities mainly owned and run by the government, which covers approximately 81.1% of the total health expenditure (THE), providing 83.1% of the hospitals, 92.5% of the hospital beds, 62.2% of all outpatient services, and 94.5% of all inpatient services [21].

### Design

A cross-sectional design was used to conduct the study. Study reporting followed the STrengthening the Reporting of OBservational studies in Epidemiology (STROBE) guidelines [22] (see Additional file 1).

### Sample and setting

This study targeted (1) adult patients and (2) all HCPs (nurses and physicians) from three departments (medical, surgical, and obstetrics and gynaecology (OBG)) at two tertiary hospitals (namely, hospitals A and B) in Oman. Data were collected over a one-month period towards the end of 2018 and the beginning of 2019. The necessary sample size for patients was estimated by power analysis, which indicated that at least 313 respondents were required for hospital ‘A’ and 158 for hospital ‘B’, where the effect size (d = 0.5), α = 0.05 and N was 6155 (4094 from hospital ‘A’ and 2061 from hospital ‘B’) discharged patients at two hospitals [21]. Patient data were collected through convenience sampling of 600 adult patients admitted to hospitals A and B (400 and 200, respectively). To minimize potential bias from convenience sampling, the authors enrolled more participants than the minimum required sampling size and maximized the participant follow-up and reminders.

The sample size for HCPs was taken from the primary study data that covered all of Oman, and HCPs were recruited though proportional stratified sampling of 246 professionals (139 nurses and 107 physicians) who worked at the two hospitals.

### Study instruments

Data for this study were collected by two items: overall quality of care and patient safety incorporated in the Revised Humane Caring Scale (RHCS) and the Healthcare Professional Core Competency Instrument (HPCCI) for patients and HCPs, respectively [23–25]. The above two items were developed by the authors and piloted as a part of the larger study with the entire RHCS and HPCCI instruments through convenience sampling of patients (*n* = 30) and HCPs (*n* = 56) at tertiary hospital in Oman. The HPCCI, that consists of 11 subscales with 81 items, was adopted from existing valid and reliable tools, and permission to use the tools was granted by their developers. The RHCS, that comprises of seven subscales with 46 items and two more items were added in this study in Oman, has been translated by experts from English to the Arabic language and backwards to English. Based on the pilot, there were no changes required to the tool. A 5-point Likert scale (1 = Failing, 2 = Poor, 3 = Acceptable, 4 = Very Good, 5 = Excellent) was used to rate the two items in the questionnaires distributed to the patients and the HCPs. The minimum score of 1 was considered to indicate failing perceptions on quality of care and patient safety while the maximum score of 5 was signifying excellent levels.

### Data collection

The principal researcher worked closely with the research assistants from the two target hospitals and explained the scope of the study and data collection process. The research assistants were given a number of questionnaires along with fact sheets; the questionnaires were distributed to both target groups: patients and HCPs, over a period of 1 month. The completed questionnaires were inserted into envelopes in locked boxes allocated to each unit. During the study period, a verbal reminder was delivered by the researcher assistants in both institutions to the target groups. The participants had the right to withdraw from the study.

### Ethical approval

Ethical approval to conduct the study was granted by the University Committee on Research Ethics (Statement 16/2018), and permission to conduct the study in the hospitals was obtained from the MOH, Sultanate of Oman (Proposal ID: MOH/CSR/18/XXXX). This study used data collected in December 2018 and January 2019. The anonymity of the participants was guaranteed, and all data were treated confidentially.

### Data analysis

Data were analysed using descriptive statistics (frequency, percentage, mean value, and standard deviation). The statistical mean was the parameter that was used to measure the overall quality of care and patient safety. A mean score of 1 indicated the lowest score, while a mean score of 5 was considered the highest. On this scale range, a mean value of 4 or more was considered ‘excellent’. This value reflects the best practices as per the literature and magnet hospital assessment scales, where 4 is defined as meeting the Magnet standards [26]. Binary logistic regression analysis was performed to determine the associations between the dependent variables (overall quality of care and patient safety) and independent variables (demographic characteristics) for both patients and HCPs. The quality of care and patient safety variables were dichotomized as combined; ‘excellent or very good’ was recorded as 1, and ‘acceptable, poor, and failing’ was recorded as 0. In this analysis, the *P* value (*P*), odds ratio (OR), and 95% confidence interval (CI) of the OR were calculated to understand how the predictors were associated with the outcomes. Multivariate and univariate analyses were performed. Data were analysed using the Statistical Package for the Social Sciences computer program (SPSS version 27.0).

## Results

### Participants’ demographic characteristics

The overall response rate for patients was 61.2% (367 of 600 targets); it corresponded to 218 patients (59.4%) from hospital A and 149 (40.6%) from hospital B. In the case of HCPs, the overall response rate was 56.9% (140 of 246 targets); there were 65 professionals (46.4%) from hospital A and 75 (53.6%) from hospital B (Table 1). Less than 30% of the patients and more than 50% of the staff fell within the group of individuals 30–40 years of age. Most of the patients and professionals were women: 58.5 and 75.5%, respectively. Most of the patients were Omani citizens (93%), and the response rate of Omani staff was slightly higher (3.6%) than that of expatriates.

**Table 1:** Participants’ demographic characteristics

Patients				Healthcare Professionals			
		n	%			n	%
**Hospital**	A	218	59.4	**Hospital**	A	65	46.4
	B	149	40.6		B	75	53.6
				**Profession**	Nurse	84	60.0
					Physician	56	40.0
**Age in (years)**	< 30	119	35.6	**Age in (years)**	< 30	28	24.6
	30–40	94	28.1		30–40	59	51.8
	> 40	121	36.2		> 40	27	23.7
**Gender**	Female	210	58.5	**Gender**	Female	105	75.5
	Male	149	41.5		Male	34	24.5
**Ethnicity**	Omani	332	93.0	**Ethnicity**	Omani	72	51.8
	Non-Omani	25	7.0		Non-Omani	67	48.2
**Living**	Alone	39	11.3	**Position**	Clinician	84	78.5
	With family	305	88.7		Management	4	3.7
**Education**	Post-secondary school education	140	40.0		Both	19	17.8
	Basic level of education	210	60.0	**Work experience**	< 8 years	41	34.2
**Occupational status**	Un-employed	154	43.9		8–15 years	44	36.7
	Employed	159	45.3		> 15 year	35	29.2
	Retiree	38	10.8	**Education**	Diploma/resident	60/13	71.4/27.1
					Bachelor/specialist	23/34	27.4/70.8
					Master/adjunct	1/0	1.2/0
					Ph.D./docent	0/1	0/2.1
**Admission area**	Medical	117	34.7	**Work area**	Medical	34	25.0
	Surgical	156	46.3		Surgical	71	52.2
	Obstetrics and gynaecology	64	19.0		Obstetrics and gynaecology	31	22.8
**Hospital admission**	Planned	132	37.7				
	Emergency	218	62.3				
**Reason of admission**	Examination	47	13.3				
	Treatment	306	86.7				
**Stay duration**	<=5 Days	192	67.6				
	> 5 Days	92	32.4				

Approximately 89% of the patients lived with their families and 60% had a basic level of education. Approximately 45% of them were employed and 44% were un-employed. Approximately 78.5% of the HCPs worked at the bedside, followed by those who had dual roles, that is, clinical and management work. There were several similarities among respondents from each working group of HCPs. Approximately two-thirds of them had between 8 and 15 years of work experience. The majority of nurses and physicians had diplomas (71.4%) and specializations (70.8%) as their educational background/qualifications.

Approximately half of the patients (46.3%) and HCPs (52.2%) were from the surgical department, followed by those from the medical department. Almost two-thirds of the patients were emergency-admitted cases (62.3%) and sought treatment rather than examination (87%). Two-thirds of the patients (67.6%) spent less than 5 days in the hospital.

### Participants’ perspectives on quality of care and patient safety

Table 2 presents the participants’ perspectives on the quality of care and patient safety standards. Overall, quality of care (patients: M = 4.23; SD = 0.706; HCPs: M = 4.36; SD = 0.720) and patient safety (patients: M = 4.22; SD = 0.709; HCPs: M = 4.39; SD = 0.675) were rated as excellent from both perspectives. However, the participants differed significantly in their views of patient safety (*p* = 0.013).

**Table 2:** Participants’ perspectives on quality of care and patient safety

Participants	Overall quality of care							Overall patient safety						
	N	M	SD	SE	P	95% CI		N	M	SD	SE	P	95% CI	
**Patients**	348	4.23	0.706	0.038	0.068	4.16	4.30	351	4.22	0.709	0.038	0.013	4.15	4.29
**HCPs**	140	4.36	0.720	0.061		4.24	4.48	140	4.39	0.675	0.057		4.28	4.50
**Total**	488	4.26	0.712	0.032		4.20	4.33	491	4.27	0.704	0.032		4.21	4.33

*N* Number of participants, *M* Mean, *SD* Standard deviation, *SE* Standard error, *P P* value, *CI* Confidence interval

### Association between demographic characteristics and overall quality of care and patient safety

A binary logistic regression analysis was performed to ascertain the association of hospital, age, gender, ethnicity, and admission/work area on the overall quality of care and patient safety. These specific variables were chosen as they feature in both instruments (RHCS and HPCCI), and a subsequent comparison can be made. Table 3 shows that patients at hospital A (OR 0.622; 95% CI 0.271–1.424; *p* = 0.261) were less satisfied with quality of care than those at hospital B, but the finding was not statistically significant. HCPs at hospital A (OR 0.095; 95% CI 0.016–0.551; *p* = 0.009) were 90% less satisfied than those at hospital B with regard to quality of care. There was also a nonsignificant tendency for men (OR 1.920; 95% CI 0.972–3.792; *p* = 0.060) to rate quality of care higher than women did. The results showed a tendency for less satisfaction with quality of care in the medical department than in the OBG department among patients (*p* = 0.036) as well as HCPs (*p* = 0.046).

**Table 3:** Binary logistic regression analysis of the quality of care

	Patients				Healthcare professionals			
	***OR***^***a***^	***CI***^***b***^ ***of OR***		***P***^***c***^	***OR***^***a***^	***CI***^***b***^ ***of OR***		***P***^***c***^
**Hospital**								
A	0.622	0.271	1.424	0.261	0.095	0.016	0.551	0.009
B	*1*	*Ref.*			1	Ref.		
**Age in (years)**								
< 30	0.860	0.408	1.813	0.692	0.131	0.010	1.707	0.121
30–40	1.901	0.755	4.791	0.173	0.148	0.014	1.606	0.116
> 40	1	Ref.		0.223	1	Ref.		0.269
**Gender**								
Male	1.920	0.972	3.792	0.060	1.496	0.255	8.790	0.656
Female	1	Ref.			1	Ref.		
**Ethnicity**								
Omani	0.571	0.166	1.967	0.375	1.941	0.420	8.962	0.396
Non-Omani	1	Ref.			1	Ref.		
**Admission/Work area**								
Medical	0.257	0.072	0.916	0.036	0.093	0.009	0.959	0.046
Surgical	0.376	0.115	1.227	0.105	0.103	0.011	0.999	0.050
Obstetrics and gynaecology	1	Ref.		0.110	1	Ref.		0.119
**Classification percentage correct**	83.3%				84.5%			
**2 Log likelihood**	241.401^a^				72.160^a^			
**Cox & Snell R Square**	.076				.185			
**Nagelkerke R Square**	.128				.321			
**Hosmer and Lemeshow**	0.528				0.338			

^a^Odds ratio

^b^95% confidence interval of odds ratio

^c^*P* value (level of significance)

Table 4 shows the results of the binary logistic regression analysis performed to assess whether demographic characteristics of patients and HCPs explain the overall perceptions of patient safety standards as good as excellent. There were no statistically significant differences between patients’ perspectives on patient safety standards at either hospital; however, patients in hospital A (OR 0.659; 95% CI 0.298–1.457; *p* = 0.303) were less satisfied than those in hospital B. Additionally, HCPs at hospital A (OR 0.153; 95% CI 0.027–0.854; *p* = 0.032) were 85% less satisfied with patient safety standards than HCPs at hospital B. There was also a nonsignificant tendency for men (OR 1.856; 95% CI 0.955–3.606; *p* = 0.068) to give better scores for patient safety standards than women. The results revealed a tendency for patients to be less satisfied with safety in the medical department than in the OBG department (*p* = 0.066).

**Table 4:** Binary logistic regression analysis of patient safety

	Patients				Healthcare professionals			
	***OR***^***a***^	***CI***^***b***^ ***of OR***		***P***^***c***^	***OR***^***a***^	***CI***^***b***^ ***of OR***		***P***^***c***^
**Hospital**								
A	0.659	0.298	1.457	0.303	0.153	0.027	0.854	0.032
B	*1*	*Ref.*			1	Ref.		
**Age in (years)**								
< 30	0.967	0.463	2.022	0.929	0.273	0.022	3.348	0.310
30–40	1.623	0.683	3.859	0.273	0.399	0.038	4.226	0.445
> 40	1	Ref.		0.445	1	Ref.		0.589
**Gender**								
Male	1.856	0.955	3.606	0.068	1.184	0.197	7.117	0.853
Female	1	Ref.			1	Ref.		
**Ethnicity**								
Omani	0.560	0.163	1.929	0.358	0.876	0.171	4.481	0.873
Non-Omani	1	Ref.			1	Ref.		
**Admission/work area**								
Medical	0.331	0.101	1.077	0.066	0.289	0.027	3.083	0.304
Surgical	0.435	0.147	1.288	0.133	0.167	0.018	1.579	0.118
Obstetrics and gynaecology	1	Ref.		0.185	1	Ref.		0.275
**Classification percentage correct**	82.3%				88.2%			
**2 Log likelihood**	254.335^a^				66.644^a^			
**Cox & Snell R Square**	.065				.114			
**Nagelkerke R Square**	.107				.220			
**Hosmer and Lemeshow**	1.000				0.249			

^a^Odds ratio

^b^95% confidence interval of odds ratio

^c^*P* value (level of significance)

## Discussion

This study had two aims: first, to examine both patients’ and HCPs’ perspectives on overall quality of care and patient safety standards at two tertiary hospitals in Oman and, second, to examine the association of demographic characteristics with the overall quality of care and patient safety. The main findings of this study indicated that quality of care and patient safety were rated relatively high, indicating competent healthcare delivery professionals and a high level of patient satisfaction.

### Perspectives on overall quality of care and patient safety

The preceding results demonstrate that patients ranked both quality of care and patient safety as excellent (4.22 and 4.23, respectively). This indicates that patients acknowledged and appreciated the healthcare services provided to them by the HCPs. This not only increases their level of satisfaction and trust in the healthcare system but may also increase their tendency to agree to treatment plans and procedures. Such a perspective may in turn help expedite patient recovery and increase the total value delivered per medical resource and intervention [27].

HCPs also ranked both quality of care and patient safety as excellent (4.39 and 4.36, respectively). This may reflect that HCPs see themselves as skilled professionals who are well-rounded in core competencies, who implement the quality assurance strategy, and who put into practice the Patient Safety Friendly Hospital Initiative (PSFHI) [4, 6].

It is worth stating that HCPs ranked themselves slightly higher in both quality of care and patient safety than did patients. This finding is consistent with Miranda et al. [28], who indicated that healthcare providers were more optimistic about their services. The following may be the reasons for this optimism: first, patients may not express their complaints regarding care because of cultural characteristics; second, HCPs may think that they provide high-quality care [29]. This finding was supported by Zhao et al. [30], who stated that nurses believed that they provided holistic care, while patients perceived that quality care may have interfered with their privacy and sleep duration.

The binary logistic regression analysis for this study showed an association of overall patient safety and quality of care with demographic characteristics (hospital, age, gender, ethnicity, and admission/work area). HCPs at hospital B rated the overall quality of care and patient safety higher than did HCPs at hospital A. This might be due to the heavier workload in hospital A because it is a specialized facility for medical and chronic cases with long durations of hospitalizations.

The findings of this study showed a significant difference in the overall quality of care among patients and HCPs in the medical department. This result matches the findings of Abuosi [31], who stated that nurses and patients had different views on quality care because they understood and characterised it differently.

This study provides meaningful insights into the perspectives of patients and HCPs on quality of care and patient safety. Such insights can be useful for current and future projects that the MOH is spearheading in line with the Sultanate’s Health Vision 2050 [41].

### Strengths and limitations

Oman has implemented quality assurance and practiced patient safety strategies at its healthcare institutions for several years, which may explain the positive findings. This should, in particular, encourage countries that have not yet implemented these strategies. However, this study has some limitations as well. First, it focused on only two variables: the overall quality of care and patient safety and their association with demographic characteristics. Second, data were collected from only three departments at two hospitals, which may affect the generalization of the study results. The response rate of both target groups, though acceptable, could have been higher [32, 33]. Third, quality of care and patient safety are broad concepts that are affected by several factors and cannot be adequately explored only through self-assessment methods. An interview and focus group discussions with patients and HCPs would therefore provide more insight into this area.

## Conclusions

This study has explored the perspectives of patients and HCPs on quality of care and patient safety in Oman. The results indicated that both patients and HCPs ranked quality of care and patient safety as excellent relative to magnet hospital standards. Thus, patients are satisfied with the levels of the healthcare delivery system and that they acknowledge and appreciate the healthcare services provided to them. This may also indicate that HCPs are well rounded in their core competencies and implement the appropriate quality assurance strategies and practices.

Hospital and admission/work area variables contributed to the overall quality of care and patient safety. These perspectives can be used to further improve delivery models at healthcare institutions in line with the Sultanate’s Health Vision 2050.

## Supplementary Information

**Additional file 1.** STROBE Statement—Checklist of Quality of Care and Patient Safety at Healthcare Institutions in Oman: Quantitative Study of the Perspectives of Patients and Healthcare Professionals.

## Data Availability

The datasets used and/or analysed during the current study are available from the corresponding author upon reasonable request.

## Abbreviations

HCPs: Healthcare professionals
CoE: Council of Europe
IOM: Institute of Medicine
WHO: World Health Organization
US: United States
MOH: Ministry of Health
PSFHI: Patient Safety Friendly Hospital Initiative
RHCS: Revised Humane Caring Scale
HPCCI: Healthcare Professional Core Competency Instrument
OBG: Obstetrics and gynaecology
N: Number of participants
M: Mean
SE: Standard error
*p*: *P* value
OR: Odds ratio
CI: Confidence interval
SPSS: Statistical Package for the Social Sciences computer program
